# Glycemic profile variability as an independent predictor of diabetic retinopathy in patients with type 2 diabetes: a prospective cohort study

**DOI:** 10.3389/fendo.2024.1383345

**Published:** 2024-11-07

**Authors:** Fatemeh Dehghani Firouzabadi, Amirhossein Poopak, Sahar Samimi, Niloofar Deravi, Pooria Nakhaei, Ali Sheikhy, Fatemeh Moosaie, Soghra Rabizadeh, Alipasha Meysamie, Manouchehr Nakhjavani, Alireza Esteghamati

**Affiliations:** ^1^ Endocrinology and Metabolism Research Center (EMRC), Vali-Asr Hospital, Tehran University of Medical Sciences, Tehran, Iran; ^2^ Department of Radiology and Imaging Sciences, Clinical Center, National Institutes of Health, Bethesda, MD, United States

**Keywords:** glycemic profile variability, HbA1c, 2hPP, FBS, diabetes type 2, diabetic retinopathy

## Abstract

**Background:**

Glycemic variability is a novel predictor for diabetic complications. Different studies have demonstrated contradictory results for the association between HbA1c variability and diabetic retinopathy. We aimed to assess the relationship between visit-to-visit variability in glycemic profile (HbA1c, 2hPP, and FBS) and diabetic retinopathy.

**Methods:**

Patients with type 2 diabetes were monitored for the development of retinopathy for 10 years. The association between the incidence of retinopathy and glycemic variability was assessed via Cox regression analysis, and coefficient of variation for glycemic indices was compared using independent sample *t*-test.

**Results:**

Patients with diabetic retinopathy had significantly higher glycemic profile variability. The incidence of retinopathy was positively correlated with cv-FBS10% (10% of coefficient of variance), cv-FBS20%, cv-2hpp10%, and cv-HbA1c5%. Our analysis revealed that the higher variability of FBS increased the incidence and progression of retinopathy (HR: 12.29, *p*-value = 0.003).

**Conclusion:**

Our findings demonstrated glycemic profile variability as an independent risk factor for diabetic retinopathy in patients with type 2 diabetes and support glycemic profile variability measurement in addition to common glycemic parameters to improve risk stratification in patients with type 2 diabetes. Further investigation is required to demonstrate the long-term effects of alleviating glycemic variability on the prognosis of patients with type 2 diabetes.

## 1 Introduction

One of the most common microvascular complications in patients with type 2 diabetes mellitus (T2DM) is diabetic retinopathy (DR), and chronic hyperglycemia is established as a main risk factor (1, 2). Recent studies have demonstrated that patients with a similar chronic glycemic profile represented by mean HbA1c may have significant differences in their short- and long-term glycemic variability (3, 4).

Fluctuations in glycemic indices, defined as “glycemic variability”, traditionally include within-day variations of patient’s glucose level as well as day-to-day fluctuations in the mean daily glucose profile, whereas long-term glycemic variability is assessed by HbA1c variability (3–5).

Based on the Diabetes Control and Complications Trial (DCCT), within-day and between-day glucose variability in patients with type 1 diabetes is not a good predictor for the initiation or progression of microvascular complications (6, 7). In contrast, HbA1c variability as a marker of long-term glucose variability was demonstrated as an independent risk for microvascular complications such as DR and diabetic nephropathy (8).

The Action in Diabetes and Vascular Disease: Preterax and Diamicron Modified Release Controlled Evaluation (ADVANCE) trial assessed the association of HbA1c and fasting blood glucose (FBS) visit-to-visit variability and major complication of diabetes. They indicated that among patients with T2DM, HbA1c variability is a predictor of macrovascular events and FBS variability is a predictor for macro- and microvascular events (9).

A cohort study on patients with T2DM from the Renal Insufficiency and Cardiovascular Events (RIACE) was led to determine the association of HbA1c variability with microvascular complications. Their analysis revealed that mean HbA1c was positively correlated with DR, whereas standard deviation (SD) for HbA1c was not (11).

In a systematic review conducted in 2015, seven studies showed a significant association between HbA1c variability and HbA1c in patients with type 1 diabetes, independent of the mean HbA1C level. In addition, 13 studies investigated HbA1c variability among patients with T2DM, which showed no association between a higher HbA1c variability and HbA1c (10).

As different studies have illustrated contradictory findings for the association between HbA1c variability and HbA1c, we aim to evaluate the association between HbA1c, FBS, and 2-h postprandial (2hPP) glucose variability with the incidence of retinopathy in patients with T2DM in this study to adopt appropriate therapeutic strategies.

## 2 Material and methods

### 2.1 Study population

In this study, patients with T2DM who were older than 18 years and attended Vali-Asr Hospital were followed up for 10 years, from February 2010 to January 2020. Each patient was visited and tested, at least four times a year (each 3 months). After excluding patients who did not complete their 10-year follow up, those who had less than four visits a year, and individuals who had a history of glaucoma, vitreous surgery, or cataract on eye examination, 294 of the 1,145 patients with T2DM developed retinopathy. This study was carried out in accordance with the latest revision of the Declaration of Helsinki, and the ethics committees of Tehran University of Medical Sciences approved the study method and protocol. The study was explained to each of the included patients and they provided written informed consent to participate in the study prior to enrollment.

### 2.2 Physical examinations

Physical examinations including measurement of height, weight, and blood pressure were performed by the trained staff. Height was measured with an inflexible measurement tape with a precision of 0.1 cm, while the subjects were asked to stand erect with no shoes and socks on. Weight was measured using a portable digital scale with a precision of 0.1 kg. Subjects were asked to wear light clothing. Body mass index (BMI) was calculated on admission by measuring height and weight (kg/m^2^). After 10 min of seated rest, trained staff measured blood pressure three times, each measured 5 min apart, using calibrated Omron M7 digital sphyg142 manometers (Hoofddorp, The Netherlands) with cuffs of appropriate size that covered at least 80% of the circumference of the right arm. The first reading was discarded. Readings from the second and third records were used to calculate the mean value for systolic and diastolic blood pressure (SBP and DBP, respectively). Waist circumference (WC) was measured by using a non-stretchable measuring tape with the patient standing still in a relaxed position, placing both feet together on a flat surface; one layer of clothing was accepted. WC was measured as the smallest horizontal girth between the costal margins and the iliac crest at minimal respiration (11). Demographic information, smoking, and medication history were obtained during the interview.

### 2.3 Laboratory evaluations

After 12–14 h of overnight fasting, 10 mL of venous blood was drawn from patients for laboratory evaluations and kept in a temperature of 4–8°C. Patients were asked to consume 75 g of glucose monohydrate powder dissolved in water, and after 2 h, another sample was drawn. All samples were sent to collaborative laboratories to centrifuge (1,500 rpm for 10 min at a standard room temperature of 21°C) in less than 4 h. The serum was extracted, stored at a temperature of −70°C, and used for laboratory evaluations. The glucose oxidase test was used to measure FBS and 2hPP with enzymatic calorimetric methods. High-performance liquid chromatography (DS5 Pink kit; Drew, Marseille, France) was used to measure HbA1c. Enzymatic methods were used to evaluate serum levels of total cholesterol (TC), low-density lipoprotein-cholesterol (LDL-c), high-density lipoprotein cholesterol (HDL-c), and triglyceride (TG). All participants were asked to collect 24-h urine samples. Urinary creatinine (Cr) excretion was measured as a gold standard to avoid false-positive results. Tests were repeated if Cr excretion levels were lower than 20 mg/kg per 24 h and 15 mg/kg per 24 h for men and women, respectively. The Jaffe method (Pars Azmun, Karaj, Iran) was used to evaluate serum Cr levels. The central reference laboratory (Tehran, Iran) tested random samples of this study for accuracy and they approved the results and the kits used in this study.

### 2.4 Assessment of diabetic retinopathy

We used the International Classification of Diseases, Tenth Revision (ICD-10), code 11.3 to screen DR. DR and the severity of DR were assessed by International Classification Level of Stereoscopic color fundus photographs from patients. The Modified Airlie House Classification was employed to determine the presence and grade of DR (12, 13).

### 2.5 Data analysis

Kolmogorov–Smirnov and Shapiro–Wilk normality tests, P–P plots, and histograms were used to test the normality of the study population. In order to determine any possible association, *t*-test and chi-square test were performed for continuous and categorical variables, respectively. The statistical significance level was assumed to be a *p*-value <0.05. The coefficient of variation (CV) of all variables for each patient was calculated. ROC curve analysis was performed to investigate sensitivity differences. The Kaplan–Meier method was used to show time-to-event and time-dependent Cox proportional hazards models. Statistics results are stated as mean ± SD for continuous variables, and as frequencies and percentages for categorical variables. All tests were performed using the SPSS software (version 24.0, Armonk, NY: IBM Corp.).

## 3 Results

### 3.1 Population characteristics

Of 1,145 patients with T2DM who participated in this study, 294 developed DR. The mean levels of the duration of diabetes (DDM), SBP, 2hPP, HbA1c, Cr, and microalbuminuria and insulin use were significantly higher in patients who developed retinopathy, during the 10-year follow up. However, no significant association was observed between the incidence of DR and the mean age, weight, WC, DBP, FBS, TC, HDL-c, LDL-c, uric acid, and BMI and there was no significant difference in the sex, smoking status, and history of hypertension between the two groups (**Table 1**).

**Table 1:** Baseline characteristics of the study population; data are presented as mean ± SD or *N* (%).

		Developed retinopathy	Did not develop retinopathy	*p*-value
**Age**		62.67 ± 10.02	61.39 ± 10.06	0.058
**Duration of diabetes**		19.95 ± 7.49	15.29 ± 6.98	<0.001
**Sex**	Male	156 (53.06%)	417 (49%)	0.230
	Female	138 (46.94%)	434 (51%)	
**Smoking**	Yes	15 (5.43%)	25 (3.16%)	0.086
	No	261 (94.57%)	767 (96.84%)	
**Insulin use**	Yes	281 (95.57%)	761 (89.42%)	0.001
	No	13 (4.43%)	90 (10.58%)	
**History of hypertension**	Yes	155 (52.72%)	404 (47.58%)	0.129
	No	139 (47.28%)	445 (52.42%)	
**Weight**		76.81 ± 16.45	78.03 ± 15.26	0.247
**Waist circumference**		99.54 ± 8.42	99.43 ± 10.06	0.863
**Systolic blood pressure**		133.48 ± 9.21	131.41 ± 14.17	0.019
**Diastolic blood pressure**		75.94 ± 4.14	76.17 ± 5.50	0.459
**FBS**	Total	151.52 ± 30.66	148.23 ± 28.32	0.093
	≤130	119.32 ± 11.13	117.78 ± 9.47	0.271
	≥130	160.66 ± 28.18	159.31 ± 24.52	0.494
**2hPP**	Total	209.13 ± 40.44	199.99 ± 38.94	0.001
	≤180	164.66 ± 12.54	156.39 ± 17.14	<0.001
	≥180	226.33 ± 33.83	219.49 ± 28.84	0.009
**HbA1c**	Total	7.85 ± 0.82	7.54 ± 0.83	<0.001
	≤7.5	7.04 ± 0.39	6.90 ± 0.46	0.003
	≥7.5	8.30 ± 0.62	8.21 ± 0.55	0.069
**Total cholesterol**		159.55 ± 30.08	157.41 ± 22.20	0.263
**HDL**		44.81 ± 8.24	45.12 ± 7.97	0.054
**LDL**		87.23 ± 19.85	86.22 ± 16.28	0.433
**TG**		144.45 ± 60.56	146.25 ± 52.70	0.627
**Creatinine**		1.16 ± 0.24	1.09 ± 0.22	<0.001
**Uric acid**		5.33 ± 1.17	5.21 ± 1.48	0.210
**Albuminuria**		103.65 ± 172.73	61.71 ± 72.91	<0.001
**BMI**		29.54 ± 6.49	29.35 ± 6.25	0.650

FBS, fasting blood sugar; 2hPP, 2-h post-prandial blood glucose level; HbA1C, hemoglobin A1c; HDL, high-density lipoprotein; LDL, low-density lipoprotein; TG, triglyceride; BMI, body mass index.

### 3.2 Retinopathy and glycemic variability

Patients who developed DR had a significantly higher CV-FBS and CV-2hpp. On further classification of FBS and 2hPP, patients who had mean FBS levels of above 130 and patients who had mean 2hPP levels above 180 and developed DR had a significantly higher CV-FBS and CV-2hPP than patients who did not develop DR, respectively. Additionally, a significantly higher CV-TC and CV-Cr was observed in patients who developed DR (**Table 2**).

**Table 2:** Coefficient of variance of the metabolic profile of the study population based on retinopathy incidence; data are presented as mean ± SD.

		Developed retinopathy	Did not develop retinopathy	*p*-value
**Weight**		0.07 ± 0.21	0.06 ± 0.18	0.646
**Systolic blood pressure**		0.10 ± 0.05	0.10 ± 0.10	0.804
**Diastolic blood pressure**		0.10 ± 0.07	0.10 ± 0.09	0.880
**FBS**	Total	0.25 ± 0.11	0.21 ± 0.11	<0.001
	≤130	0.24 ± 0.12	0.21 ± 0.11	0.102
	≥130	0.26 ± 0.10	0.22 ± 0.11	<0.001
**2hPP**	Total	0.26 ± 0.10	0.24 ± 0.10	<0.001
	≤180	0.28 ± 0.11	0.23 ± 0.11	0.053
	≥180	0.26 ± 0.10	0.24 ± 0.10	0.002
**HbA1c**	Total	0.11 ± 0.09	0.10 ± 0.09	0.420
	≤7.5	0.11 ± 0.03	0.10 ± 0.05	0.118
	≥7.5	0.11 ± 0.11	0.10 ± 0.13	0.759
**Total cholesterol**		0.18 ± 0.18	0.15 ± 0.06	0.002
**HDL-c**		0.15 ± 0.07	0.14 ± 0.06	0.060
**LDL-c**		0.24 ± 0.09	0.23 ± 0.11	0.565
**TG**		0.28 ± 0.11	0.27 ± 0.12	0.217
**Creatinine**		0.21 ± 0.12	0.19 ± 0.11	0.003
**Uric acid**		0.18 ± 0.19	0.16 ± 0.15	0.171
**Albuminuria**		0.73 ± 0.31	0.69 ± 0.33	0.108

FBS, fasting blood sugar; 2hPP, 2-h post-prandial blood glucose level; HbA1C, hemoglobin A1c; HDL, high-density lipoprotein; LDL, low-density lipoprotein; TG, triglyceride; BMI, body mass index.

**Figure 1:**
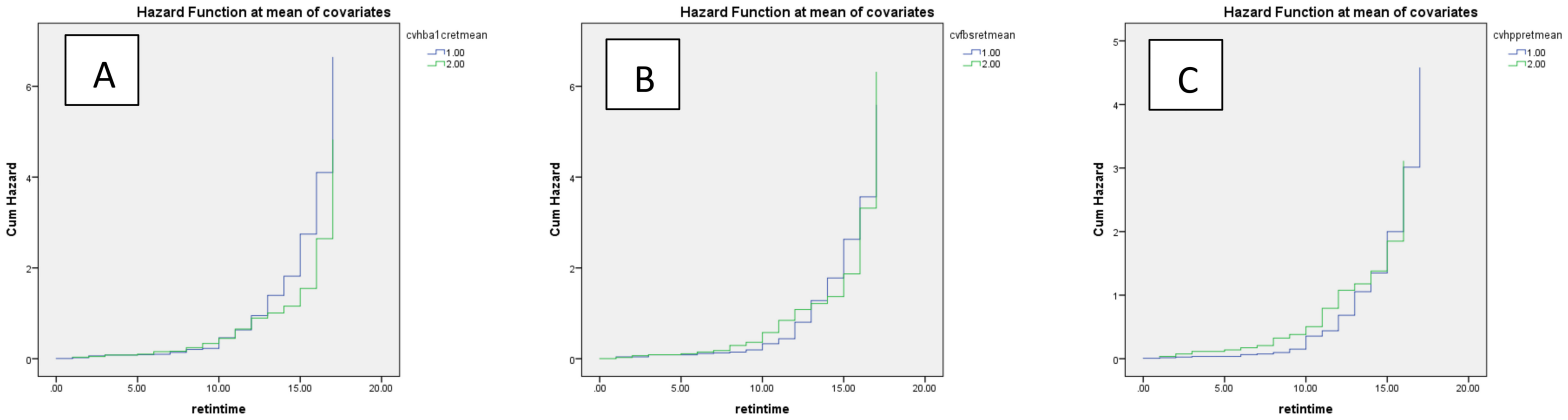
Cumulative hazard of retinopathy based on coefficient of variance of glycemic indices **(A)** HbA1c, **(B)** FBS, and **(C)** 2hpp during the follow-up period. Blue and green lines indicate values above and below the mean, respectively.

**Figure 2:**
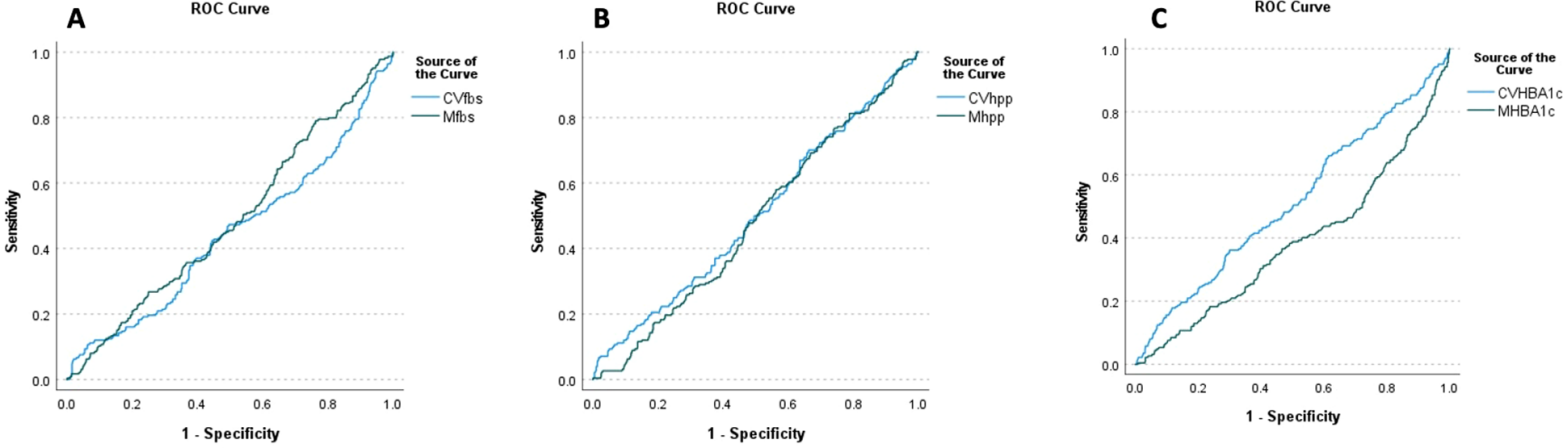
ROC curve analysis of retinopathy based on the mean and coefficient of variance of glycemic indices **(A)** HbA1c, **(B)** FBS, and **(C)** 2hpp during the follow-up period. FBS (AUC difference = −0.43, *p*-value = 0.146), 2hpp (AUC difference = 0.493, *p*-value = 0.020), and HbA1c (AUC difference = 1.27, *p*-value < 0.001).

In the multivariate Cox regression model, a significant positive association between CV-FBS and the incidence of DR was revealed (HR: 12.289, *p*-value = 0.003). CV-TC, CV-LDL, CV-HDL, and CV-Cr were positively associated with the incidence of DR (*p*-value = 0.003, *p*-value = 0.042, *p*-value = 0.024, and *p*-value = 0.019, respectively). A significant positive correlation between patients who developed DR and CV-FBS10% and CV-FBS20% levels was revealed (*p*-value = 0.003 and *p*-value ≤ 0.001, respectively). Patients who developed DR were positively correlated with CV-2hPP10% and CV-HbA1c5% (*p*-value = 0.039 and *p*-value = 0.009, respectively) (**Table 3**) (**Figure 1**). Our ROC curve analysis showed a significant difference in the area under the curve between mean and coefficient of variances in 2hpp and HbA1c [FBS (AUC difference = −0.43, *p*-value = 0.146), 2hpp (AUC difference = 0.493, *p*-value = 0.020), and HbA1c (AUC difference = 1.27, *p*-value < 0.001)] (**Figure 2**).

**Table 3:** Multivariate Cox regression model determining the association between glycemic indices variability and incidence of retinopathy.

	*p*-value	Hazard ratio	95% confidence interval	
**CV-FBS**	0.003	12.289	2.359	64.008
**CV-2HPP**	0.467	0.536	0.100	2.878
**CV-HbA1c**	0.095	4.770	0.763	29.823
**CV-cholesterol**	0.003	36.592	3.354	399.245
**CV-HDL**	0.024	13.659	1.400	133.290
**CV-LDL**	0.042	1.138	1.021	1.934
**CV-TG**	0.882	0.909	0.258	3.205
**CV-SBP**	0.919	1.090	0.206	5.769
**CV-DBP**	0.548	0.512	0.058	4.552
**CV-Creatinine**	0.019	4.238	1.262	14.234
**CV-Albuminuria**	0.348	1.228	0.800	1.887
**CV-Uric acid**	0.284	1.546	0.696	3.431
**CV-weight**	0.805	1.094	0.538	2.223
Association between incidence of retinopathy and glycemic indices variability				
	Odds ratio	95% confidence interval	*p*-value	
**CVFBS5%**	1.036	0.108–9.925	0.975	
**CVFBS10%**	2.522	1.320–4.819	0.003	
**CVFBS20%**	1.454	1.232–1.715	<0.001	
**CV2HPP5%**	0.999	0.997–1.001	0.557	
**CV2HPP10%**	3.224	1.988–10.528	0.039	
**CV2HPP20%**	1.157	0.955–1.401	0.129	
**CVHBA1c5%**	1.981	1.163–3.372	0.009	
**CVHBA1c10%**	1.115	0.978–1.271	0.093	
**CVHBA1c20%**	0.996	0.975–1.017	0.689	

FBS, fasting blood sugar; 2hpp, 2-h post-prandial blood glucose level; HbA1C, hemoglobin A1c; HDL, high-density lipoprotein; LDL, low-density lipoprotein; TG, triglyceride; BMI, body mass index.

## 4 Discussion

In this prospective cohort study with a median follow-up of 10 years, we focused on the visit-to-visit variability of FBS, 2hPP, and HbA1c. This study demonstrated that patients who developed DR had significantly higher FBS variability with a cutoff of 10% for CV-FBS. Moreover, we provided evidence that higher variability of FBS increases the risk of DR incidence (HR: 12.29), proving glycemic profile variability as an independent risk factor for DR in patients with T2DM.

The findings of previous studies are mostly in line with ours. Cardoso et al. evaluated the prognostic value of several measures of glycemic variability for the incidence of microvascular complications in Brazilian adults with T2DM over nearly 10 years of follow-up. According to their findings, visit-to-visit glycemic variability particularly the 24-month parameters estimated by either FBS or HbA1c could predict retinopathy development and progression in patients with good glycemic control (HbA1c ≤ 7.5%) (14). Hsieh et al. recently reported that high visit-to-visit FBS variability was associated with the incidence of proliferative DR and diabetic macular edema, independent of mean and baseline FBS and HbA1c levels in patients with T2DM. However, HbA1c variability (calculated as SD and CV) was not associated with the development of DR in their results (15). Moreover, Lu et al., among a total of 3,262 patients with T2DM, showed that time in range (defined as the percentage of a 24-h period that the glucose level stayed between 3.9 and 10.0 mmol/L) was significantly associated with all stages of DR (16). A recent meta-analysis also reported that high FBS variability (evaluated by mean or median FBS variability levels) was strongly associated with the risk of DR [odds ratio (OR) = 3.68; 95% CI 1.01–13.4] (17).

Several reports have assessed the pathophysiology underlying the association between glycemic variability and DR. Firstly, transient high glucose level spikes in addition to persistent chronic hyperglycemia may cause epigenetic changes due to higher oxidative stress (18), which also increases insulin resistance and pancreatic β-cell dysfunction and apoptosis (19, 20), all known possible risk factors for DR incidence (21, 22). Secondly, glucose fluctuations can damage endothelial function in microvascular beds (23). Therefore, oscillating glucose levels may lead to endothelial dysfunction, hence change the morphology of vessel walls more than a consistently high glucose concentration (24, 25). Interestingly, Costantino et al. reported that mean glycemic excursions were independently associated with adverse epigenetic signatures on p66^Shc^ promoter, which induced chromatin changes, leading to persistent vascular dysfunction in individuals with T2DM and with HbA1c levels in the target range (26). A 2021 study by Saik et al. reconstructed and analyzed the gene networks related to glucose variability in T2DM and its complications. They reported that glycemic variability-related genes occupied central positions in the network of diabetes-associated complications including DR and were associated with response to hypoxia (27). These results further confirm the previously mentioned mechanisms underlying the association between glycemic variability and DR.

The present study had some limitations that shall be addressed. Firstly, it is a prospective cohort study; therefore, neither cause-and-effect relations, nor pathophysiological deductions, could be made, but only speculated. Secondly, residual confounding effect due to unknown or unmeasured factors could not be ruled out. Thirdly, individuals not adherent to follow-up visits were excluded from the study, and this may have excluded those at greater risk of poor glycemic control and DR from the study. Fourthly, since the case number of new-onset DR was relatively small, the statistical power could be low for examining the risk factors, although those factors that were significant held their validity. On the other hand, the current study had several strengths. The main strength is it being a well-documented, large-scale cohort with standardized care as well as annual outcome evaluations over a long follow-up, permitting a comprehensive analysis of the associations between long-term glycemic profile variability and risk of DR. Moreover, to the best of our knowledge, this is the first study in the Middle East and North Africa (MENA) region to investigate the association between glycemic profile variability and retinopathy incidence in patients with T2DM.

## 5 Conclusion

This large-scale prospective cohort study with a 10-year follow-up of patients with T2DM provides evidence that glycemic profile variability is an independent risk factor for DR. Therefore, to improve risk stratification in patients with T2DM, our findings support glycemic profile variability measurement in addition to common glycemic parameters. However, future studies are necessary to demonstrate whether this reduction translates into better prognosis for patients with T2DM. Moreover, further studies are needed to elucidate the impact of glycemic variability on the incidence and progression of DR and its effect in regard to various treatment modalities for DR.

## Data Availability

The raw data supporting the conclusions of this article will be made available by the authors, without undue reservation.

## References

[1] Lachin JM Genuth S Nathan DM Zinman B Rutledge BN DCCT/EDIC Research Group. Effect of glycemic exposure on the risk of microvascular complications in the diabetes control and complications trial—revisited. Diabetes. (2008) 57(4):995–1001. doi: 10.2337/db07-1618 18223010

[2] Cheung N Mitchell P Wong TY. Diabetic retinopathy. Lancet. (2010) 376:124–36. doi: 10.1016/S0140-6736(09)62124-3 20580421

[3] Kilpatrick EJD. The rise and fall of HbA 1c as a risk marker for diabetes complications. Diabetologia. (2012) 55(8):2089–91. doi: 10.1007/s00125-012-2610-5 22711013

[4] Moosaie F Mouodi M Sheikhy A Fallahzadeh A Deravi N Rabizadeh S. Association between visit-to-visit variability of glycemic indices and lipid profile and the incidence of coronary heart disease in adults with type 2 diabetes. J Diabetes Metab Disord. (2021) 20:1715–23. doi: 10.1007/s40200-021-00930-z PMC8630209 34900821

[5] Firouzabadi MD Poopak A Sheikhy A Samimi S Nakhaei P Firouzabadi FD. Glycemic profile variability: An independent risk factor for diabetic neuropathy in patients with type 2 diabetes. Primary Care Diabetes. (2023) 17:38–42. doi: 10.1016/j.pcd.2022.11.011 36464622

[6] Kilpatrick ES Rigby AS Atkin SL. The effect of glucose variability on the risk of microvascular complications in type 1 diabetes. Random Contr Trial. (2006) 29(7):1486–90. doi: 10.2337/dc06-0293 16801566

[7] Kilpatrick ES Rigby AS Atkin SL. Effect of glucose variability on the long-term risk of microvascular complications in type 1 diabetes. Diabetes Care. (2009) 32(10):1901–3. doi: 10.2337/dc09-0109 PMC2752912 19549736

[8] Kilpatrick ES Rigby AS Atkin SL. A1C variability and the risk of microvascular complications in type 1 diabetes: data from the Diabetes Control and Complications Trial. Diabetes Care. (2008) 31(11):2198–202. doi: 10.2337/dc08-0864 PMC2571045 18650371

[9] Hirakawa Y Arima H Zoungas S Ninomiya T Cooper M Hamet P. Impact of visit-to-visit glycemic variability on the risks of macrovascular and microvascular events and all-cause mortality in type 2 diabetes: the ADVANCE trial. Diabetes Care. (2014) 37:2359–65. doi: 10.2337/dc14-0199 24812434

[10] Gorst C Kwok CS Aslam S Buchan I Kontopantelis E Myint PK. Long-term glycemic variability and risk of adverse outcomes: a systematic review and meta-analysis. Diabetes Care. (2015) 38:2354–69. doi: 10.2337/dc15-1188 26604281

[11] Esteghamati A Ismail-Beigi F Khaloo P Moosaie F Alemi H Mansournia MA. Determinants of glycemic control: Phase 2 analysis from nationwide diabetes report of National Program for Prevention and Control of Diabetes (NPPCD-2018). Primary Care Diabetes. (2020) 14(3):222–31. doi: 10.1016/j.pcd.2019.07.002 31402326

[12] Dugan J Shubrook J. International classification of diseases, 10th revision, coding for diabetes. Clin diabetes: Publ Am Diabetes Assoc. (2017) 35:232–8. doi: 10.2337/cd16-0052 PMC5669129 29109613

[13] Wu L Fernandez-Loaiza P Sauma J Hernandez-Bogantes E Masis M. Classification of diabetic retinopathy and diabetic macular edema. World J Diabetes. (2013) 4:290–4. doi: 10.4239/wjd.v4.i6.290 PMC3874488 24379919

[14] Cardoso C Leite NC Moram CBM Salles GF. Long-term visit-to-visit glycemic variability as predictor of micro-and macrovascular complications in patients with type 2 diabetes: the Rio de Janeiro Type 2 Diabetes Cohort Study. Cardiovasc Diabetol. (2018) 17:1–16. doi: 10.1186/s12933-018-0677-0 29477146 PMC6389075

[15] Hsieh YT Hsieh MC. Fasting plasma glucose variability is an independent risk factor for diabetic retinopathy and diabetic macular oedema in type 2 diabetes: An 8-year prospective cohort study. Clin Exp Ophthalmol. (2020) 48:470–6. doi: 10.1111/ceo.13728 32065699

[16] Lu J Ma X Zhou J Zhang L Mo Y Ying L. Association of time in range, as assessed by continuous glucose monitoring, with diabetic retinopathy in type 2 diabetes. Diabetes Care. (2018) 41:2370–6. doi: 10.2337/dc18-1131 30201847

[17] Zhao Q Zhou F Zhang Y Zhou X Ying C. Fasting plasma glucose variability levels and risk of adverse outcomes among patients with type 2 diabetes: a systematic review and meta-analysis. Diabetes Res Clin Pract. (2019) 148:23–31. doi: 10.1016/j.diabres.2018.12.010 30583033

[18] El-Osta A Brasacchio D Yao D Pocai A Jones PL Roeder RG. Transient high glucose causes persistent epigenetic changes and altered gene expression during subsequent normoglycemia. J Exp Med. (2008) 205:2409–17. doi: 10.1084/jem.20081188 PMC2556800 18809715

[19] Groop P-H Forsblom C Thomas MC. Mechanisms of disease: pathway-selective insulin resistance and microvascular complications of diabetes. Nat Clin Pract Endocrinol Metab. (2005) 1:100–10. doi: 10.1038/ncpendmet0046 16929378

[20] Del Guerra S Grupillo M Masini M Lupi R Bugliani M Torri S. Gliclazide protects human islet beta-cells from apoptosis induced by intermittent high glucose. Diabetes/metabolism Res Rev. (2007) 23:234–8. doi: 10.1002/dmrr.v23:3 16952202

[21] Calderon G Juarez OH Hernandez GE Punzo SM De la Cruz ZD. Oxidative stress and diabetic retinopathy: development and treatment. Eye. (2017) 31:1122–30. doi: 10.1038/eye.2017.64 PMC5558229 28452994

[22] Li Y-Y Yang XF Gu H Snellingen T Liu XP Liu NP. The relationship between insulin resistance/β-cell dysfunction and diabetic retinopathy in Chinese patients with type 2 diabetes mellitus: the Desheng Diabetic Eye Study. Int J Ophthalmol. (2018) 11:493. doi: 10.18240/ijo.2018.03.21 29600185 PMC5861241

[23] Zhang XG Zhang YQ Zhao DK Wu JX Zhao J Jiao XM. Relationship between blood glucose fluctuation and macrovascular endothelial dysfunction in type 2 diabetic patients with coronary heart disease. Eur Rev Med Pharmacol Sci. (2014) 18:3593–600. 25535128

[24] Ceriello A Esposito K Piconi L Ihnat MA Thorpe JE Testa R. Oscillating glucose is more deleterious to endothelial function and oxidative stress than mean glucose in normal and type 2 diabetic patients. Diabetes. (2008) 57:1349–54. doi: 10.2337/db08-0063 18299315

[25] Schisano B Tripathi G McGee K McTernan PG Ceriello A. Glucose oscillations, more than constant high glucose, induce p53 activation and a metabolic memory in human endothelial cells. Diabetologia. (2011) 54:1219–26. doi: 10.1007/s00125-011-2049-0 21287141

[26] Costantino S Paneni F Battista R Castello L Capretti G Chiandotto S. Impact of glycemic variability on chromatin remodeling, oxidative stress, and endothelial dysfunction in patients with type 2 diabetes and with target HbA1c levels. Diabetes. (2017) 66:2472–82. doi: 10.2337/db17-0294 28634176

[27] Saik OV Klimontov VV. Bioinformatic reconstruction and analysis of gene networks related to glucose variability in diabetes and its complications. Int J Mol Sci. (2020) 21:8691. doi: 10.3390/ijms21228691 33217980 PMC7698756

